# Management practices for West syndrome in South Asia: A survey study and meta‐analysis

**DOI:** 10.1002/epi4.12419

**Published:** 2020-08-11

**Authors:** Priyanka Madaan, Prem Chand, Kyaw Linn, Jithangi Wanigasinghe, Mimi Lhamu Mynak, Prakash Poudel, Raili Riikonen, Amit Kumar, Pooja Dhir, Sandeep Negi, Jitendra Kumar Sahu

**Affiliations:** ^1^ Pediatric Neurology Unit Department of Pediatrics Postgraduate Institute of Medical Education and Research Chandigarh India; ^2^ Aga Khan University Karachi Pakistan; ^3^ Pediatric Neurology Unit Yangon Children Hospital Yangon Myanmar; ^4^ Department of Paediatrics University of Colombo Colombo Sri Lanka; ^5^ Department of Pediatrics Jigme Dorji Wangchuck National Referral Hospital Thimphu Bhutan; ^6^ Department of Pediatrics B.P. Koirala Institute of Health Sciences Dharan Nepal; ^7^ Child Neurology Children's Hospital University of Eastern Finland and Kuopio University Hospital Kuopio Finland; ^8^ Department of Neurology All India Institute of Medical Sciences New Delhi India

**Keywords:** Asia, epileptic spasms, hypsarrhythmia, infantile spasms, low middle‐income countries

## Abstract

**Objectives:**

Considering the dearth of literature on West syndrome (WS) from South Asian countries, this study aimed to evaluate the management practices in South Asia by an online survey and meta‐analysis.

**Methods:**

An online questionnaire was sent to 223 pediatric neurologists/pediatricians in India, Pakistan, Myanmar, Sri Lanka, Bhutan, Nepal, and Bangladesh. Their responses were evaluated and supplemented by a meta‐analysis.

**Results:**

Of 125 responses received (response rate: 56%), around 60% of responders observed male preponderance and an approximate lead‐time‐to‐treatment (LTTT) of 4‐12 weeks. The commonest etiology observed was a static structural insult (88.6% of responders). Most commonly used first‐line drug (country‐wise) was as follows: India—adrenocorticotropin hormone (ACTH, 50%); Pakistan—oral steroids (45.5%); Myanmar, Sri Lanka, and Nepal—oral steroids (94.4%); Bangladesh—ACTH (2/2); Bhutan—vigabatrin (3/5). ACTH and vigabatrin are not available in Myanmar and Nepal. The most commonly used regime for ACTH was maximal‐dose‐at‐initiation‐regime in India, Sri Lanka, and Bangladesh and gradually escalating‐regime in Pakistan. Maximum dose of prednisolone was variable—most common response from India: 3‐4 mg/kg/d; Pakistan, Bhutan, and Bangladesh: 2 mg/kg/d; Sri Lanka, Nepal, and Myanmar: 5‐8 mg/kg/d or 60 mg/d. The total duration of hormonal therapy (including tapering) ranged from 4 to 12 weeks (67/91). Most responders considered cessation of spasms for four weeks as complete response (54/111) and advised electroencephalography (EEG; 104/123) to check for hypsarrhythmia resolution. Difficult access to pediatric EEG in Bhutan and Nepal is concerning. More than 95% of responders felt a need for more awareness. The meta‐analysis supported the preponderance of male gender (68%; confidence interval [CI]: 64%‐73%), structural etiology(80%; CI 73%‐86%), longer LTTT (2.4 months; CI 2.1‐2.6 months), and low response rate to hormonal therapy(18% and 28% for ACTH and oral steroids respectively) in WS in South Asia.

**Significance:**

This study highlights the practices and challenges in the management of WS in South Asia. These include a preponderance of male gender and structural etiology, a longer LTTT, difficult access to pediatric EEG, nonavailability of ACTH and vigabatrin in some countries, and low effectiveness of hormonal therapy in this region.

**Key Points**

- There is preponderance of male gender and structural etiology in South Asian children with West syndrome.
- The response rates to hormonal therapy in WS are low in South Asia
- Difficult access to pediatric EEG and nonavailability of ACTH and vigabatrin are the other problems in some of this region.
- There is a growing fondness for oral steroids in WS in South Asia.

## 1 INTRODUCTION

West syndrome (WS), the commonest epileptic encephalopathy in infancy, is characterized by clustered epileptic spasms and a pathognomonic electroencephalographic (EEG) pattern of hypsarrhythmia.^1^ Infantile spasms (IS) and WS are often used synonymously but IS also encompasses epileptic spasms with or without a cluster, and EEG abnormalities (even without hypsarrhythmia). WS/IS comprises 2% of childhood epilepsies and 13%‐45% of infantile‐onset epilepsies.^2^, ^3^, ^4^ These may be caused by an array of etiologies, including structural, metabolic, and genetic variations.

Diagnosis of WS warrants early and aggressive treatment because early control of epileptic spasms and shorter lead‐time‐to‐treatment (LTTT) have been associated with better outcomes, especially in cases with unknown etiology.^5^ The therapeutic options include vitamin trials (pyridoxine, pyridoxal phosphate, and folinic acid in cryptogenic WS), hormonal therapy [intramuscular adrenocorticotrophic hormone (ACTH)/ oral steroids], and vigabatrin. ACTH (level B) or vigabatrin (level C) may be advised as the first‐line treatment of WS and ACTH has an edge on vigabatrin (level C).^2^, ^6^ Besides these first‐line options, there is growing literature available on other drugs such as zonisamide and topiramate.^7^, ^8^

A recent meta‐analysis estimated the pooled incidence of WS/IS to be 0.249 cases/1000 live births with a pooled prevalence of 0.015 cases/1000 population and a higher prevalence in Scandinavian countries.^9^

However, the included studies were predominantly from North America, Europe, and parts of Asia (Japan, Taiwan, South Korea, Kazakhstan, China, Singapore, and India). The underrepresentation of African and Asian continent in this review strongly implies a dearth of literature on WS/IS from these countries. Also, there are several other crucial facets that need to be addressed in the management of WS. These include frequent misdiagnosis, diagnostic and treatment lag, availability of and familiarity with treatment modalities, quantification of response to treatment, and neurodevelopmental sequelae.^10^, ^11^ These fronts are handled differently in not only different clinical setups but also in different nations.^12^, ^13^, ^14^

There are several known disparities in the practices for the management of WS in Asia as compared with Europe and North America. These include poor access to pediatric neurology services, preponderance of structural etiology, nonavailability of ACTH and vigabatrin in some countries, cost of vigabatrin, increased risk of tropical infections, and constraint of resources.^13^, ^14^ Also, many of the South Asian countries lack published literature on their practices, experience, and problems. Hence, the ground reality of some of these countries is not known. Therefore, the current study aimed to capture the prevailing practices for the management of WS in South Asia by means of an online survey. The secondary objective was to carry out a meta‐analysis of the existing literature covering this aspect.

## 2 METHODS

The descriptive, cross‐sectional survey study was designed at a tertiary care hospital in northern India. This study was conducted over seven months (March to September 2019) after approval from the Institutional Ethics Committee (IEC/2018/002183). A questionnaire consisting of 36 questions was prepared. The questions pertained to (a) responders' demographics and qualification, (b) epidemiological variables (gender preponderance, etiology of WS), (c) diagnosis of WS (diagnostic modalities used, treatment lag, and its causes), (d) treatment (preferred order of treatment, availability and licensing of first‐line drugs, treatment regimen followed for hormonal therapy, and vigabatrin), (e) response assessment (clinical and electroencephalographic) in responder's clinical setting. Although the response to a few questions was compulsory, responding to each question was not mandatory to proceed to the next question. This was done to get a better response rate.

An online survey was designed using Google forms. The link to the survey was shared via emails with a total of 223 pediatric neurologists/pediatricians in India (165), Pakistan (22), Myanmar (11), Bhutan (10), Sri Lanka (8), Nepal (5), and Bangladesh (2). In India, the mailing list of the pediatric neurologists and pediatricians with a special interest in neurology who are part of the Association of Child Neurology (AOCN), India, was primarily used for the survey. A coinvestigator was identified at Pakistan, Nepal, Bhutan, Sri Lanka, and Myanmar who shared the survey link with the practitioners managing WS in their respective country. However, a coinvestigator could not be identified in Bangladesh, and the survey link was shared with only two pediatric neurologists with already known contact details. The nonresponders were encouraged by frequent emails. The responses were analyzed by using Statistical Package for Social Sciences, version 22 software (IBM Corporation, Armonk, NY, USA).

The existing literature from South Asia was systematically reviewed and a meta‐analysis for several variables (gender, etiology, lead time to diagnosis/treatment (LTTD/LTTT), and response rate to ACTH and oral steroids, respectively) was done to authenticate the results of the survey. Preferred reporting items for systematic reviews and meta‐analyses (PRISMA) diagram and detailed methodology are illustrated in Figure S1 and Table S1.

## 3 RESULTS

### 3.1 Results of the survey

**FIGURE 1:**
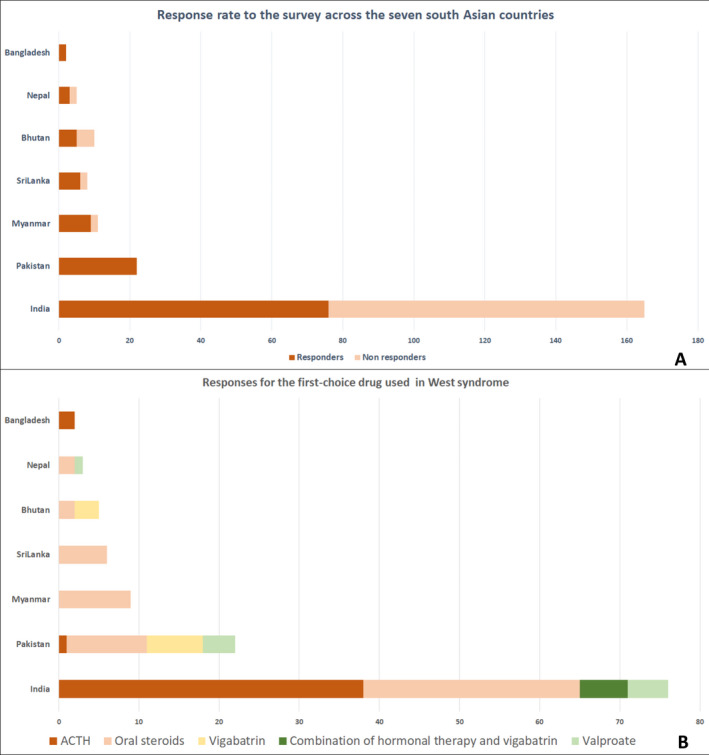
Country‐wise survey response rate and preferred first‐choice drug for West syndrome in South Asia. Bar diagram showing the response rate to the survey across the seven South Asian countries (A) and responses for the first‐choice drug used in West syndrome (B)

One hundred twenty‐five responses were received [response rate: 56%; India (76/165), Pakistan (22/22), Myanmar (9/11), Sri Lanka (6/8), Bhutan (5/10), Nepal (3/5), Bangladesh (2/2), and outliers (2/165; UK and UAE); Figure 1A]. Most responders from India (53/76), Pakistan (18/22), Myanmar (7/9), Sri Lanka (6/6), and Nepal (2/3) were qualified pediatric neurologists/epileptologists. The key responses are demonstrated in Table 1.

**TABLE 1:** Responses of pediatricians/ pediatric neurologists managing West syndrome to the survey questions

Question (number of responses received)	India	Pakistan	Myanmar	Sri Lanka	Bhutan	Nepal	Bangladesh	Total n (%)
Number of responses for each country	76	22	9	6	5	3	2	123
Qualification of responders (123)								
MD/ DNB Pediatrics with experience	23	4	2	0	5	1	1	40 (32.5%)
Qualified in neurology/ epilepsy	53	18	7	6	—	2	1	83 (67.5%)
Male preponderance observed (123)	45	14	4	4	3	2	2	74 (60.2%)
Approximate lead time to treatment (123)								
<4 w	11	2	—	4	1	1	—	19 (15.4%)
4 w to 3 mo	45	10	8	2	3	2	1	71 (57.7%)
3 mo to 6 mo	18	6	1	—	1	—	1	27 (22%)
>6 mo	2	4	—	—	—	—	—	6 (4.9%)
Common causes of treatment lag (123; multiple)								
Lack of recognition as seizure by parents	72	15	9	6	4	3	2	111(90.2%)
Poor accessibility to medical facility	23	8	4	1	1	1	2	40 (32.5%)
Misdiagnosis by attending physician	57	13	5	3	3	3	1	85 (69.1%)
Lack of EEG facility	10	2	0	0	5	2	1	20 (16.3%)
Time taken for investigations EEG/MRI	15	8	0	0	3	2	2	30 (24.4%)
Provision for free diagnosis and treatment of children with West syndrome(123)	32	9	7	6	5	1	0	60 (48.8%)
What is the most common cause of West syndrome in your practice? (122)								
Static insult sequelae: Hypoxic‐ischemic/ Hypoglycemic brain injury etc	73	14	9	6	(Response: 4/5) 2	3	1	108 (88.6%)
Tuberous Sclerosis Complex	—	—	—	—	2	—	—	2 (1.6%)
Inborn errors of metabolism	1	1	—	—	—	—	—	2 (1.6%)
Genetic causes	2	7	—	—	—	—	1	10 (8.2%)
First choice of drug used (123)								
ACTH IM	38	1	—	—	—	—	2	41 (33.4%)
ACTH + Vigabatrin	5	—	—	—	—	—	—	5 (4.1%)
Oral steroids	27	10	9	6	2	2	—	56 (45.5%)
Oral valproate	5	4	—	—	—	1	—	10 (8.1%)
Oral vigabatrin	—	7	—	—	3	—	—	10 (8.1%)
Steroid + vigabatrin	1	—	—	—	—	—	—	1 (0.8%)
What duration of cessation of spasms is defined as a complete therapeutic response? (111)	4 w (33/ 68), 2 w (30/68), 1 w (5/68)	4 w (12/20), 2 w (4/20), 1 w (3/20), 4‐6 w (1/20)	4 w (3/9), 2 w (4/9), 6 w (1/9), 1 w (1/9)	4 w (2/5), 2 w (2/5), 1 w (1/5)	4 w (3/4), 2 w (1/4)	4 w (1/ 3), 2 w (2/3)	2 w (2/2)	4 w (54; 48.6%), 2 w (46; 41.5%)
EEG used to document resolution of hypsarrhythmia (123)	68	18	7	6	0	3	2	104 (84.6%)
When do you proceed for the second‐line drug after failure of clinical response to first‐line drug? (121)	2 w (43/75), 4 w (16/75), Others (16/75)	2 w (10/22), 4 w (8/22), Others (4/ 22)	2 w (9/9)	2 w (4/6), 4 w (2/6)	2 w (2/4), 4 w (2/4)	2 w (3/3)	2 w (2/2)	2 w (73; 60.3%), 4 w (28; 23.1%)
An established Standard Operating Protocol followed in practice (123)	49	2	8	3	0	0	0	62 (50.4%)
A felt need for more awareness (123)	75	22	9	5	5	3	2	121 (98.4%)

Abbreviations: ACTH, adrenocorticotrophic hormone; EEG, electroencephalogram; IM, intramuscular; mo, months; MRI, Magnetic Resonance Imaging; w, weeks.

Around 60% of responders (74/123) observed gender preponderance for males in their experience. Treatment lag was fairly common in the South Asian subcontinent. Most responders (71/123; 58%) suggested that approximate LTTT in their respective countries ranged from 4 to 12 weeks. However, some regional differences were noted, such as most responders from Sri Lanka (4/6) observing a shorter LTTT of fewer than four weeks. Most responders felt that the commonest cause of treatment lag was lack of event recognition as a seizure by parents (111/123; 90%) followed by misdiagnosis by attending physician (85/123; 69%). Besides these, a lack of pediatric EEG facilities in Bhutan (5/5) and Nepal (2/3) is a matter of concern. Most responders from Bhutan, Sri Lanka, and Myanmar had provision for free diagnosis and management of children with WS while this was applicable for around 40% responders from India, Pakistan, and Nepal.

Most responders (88.6%) considered static structural insult (such as hypoxic‐ischemic encephalopathy, and hypoglycemic brain injury) as the commonest cause of WS in their practice. Around 30% responders from Pakistan had observed genetic causes of WS more commonly as compared with structural insult. For WS with unknown etiology, most responders would proceed with either metabolic workup (first choice: 64/123; second choice: 44/123) or pyridoxine trial (first choice: 57/123; second choice: 3/123) or vice versa. Some responders would straightaway go ahead with genetic testing—chromosomal microarray (6/123), epileptic encephalopathy panel (8/123), and exome sequencing (7/123).

Hormonal therapy (ACTH or oral steroids) was the first‐line treatment used by most responders (97/123; 78.9%). In India, 38/76 (50%) use ACTH, 27/76 (35.5%) responders use oral steroids, 6/76 (8%) use a combination therapy (hormonal + vigabatrin), and 5/76 (7%) use valproate as the first drug for WS (Figure 1B). In Pakistan, the responders use oral steroids (10/22; 45.5%), vigabatrin (7/22; 31.8%%), and valproate (4/22; 18.2%) as the first‐line drug. ACTH as the first‐line therapy was chosen minimally (1/22). In Myanmar, Sri Lanka, and Nepal, oral steroids (17/18) were the preferred for first‐ line use. In Bangladesh, ACTH was preferred (2/2) while in Bhutan, oral steroids (2/5) and vigabatrin (3/5) were equally chosen.

More than 75% of responders from Pakistan, India, and Bangladesh used ACTH in their practice. However, it was not at all used by responders from Myanmar, Nepal, and Bhutan. The most commonly used preparation of ACTH was Acton Prolongatum (most responders for this question were from India–44/56) (Table 2). The most commonly used regimen was maximal‐dose‐at‐initiation followed by tapering in India, Sri Lanka, and Bangladesh while in Pakistan, most responders followed a gradually escalating regimen with once‐daily administration. For maximal‐dose‐at‐initiation regimen, the initial dose ranged from 60‐75 IU/day of ACTH. In Sri Lanka, responders followed an alternate day regime of ACTH. ACTH was mostly used in combination with other antiepileptic drugs (68/81).

**TABLE 2:** Management practices pertaining to use of hormonal therapy in West syndrome in the surveyed countries

Question (Responses received)	India	Pakistan	Myanmar	Sri Lanka	Bhutan	Nepal	Bangladesh
ACTH used in practice (123)	65/76	17/22	0/9	2/6	0/5	0/3	2/2
Most common preparation of ACTH used (56)	Acton Prolongatum (41/50); Others: (9/50)	Acton Prolongatum (2/4); Others:(2/4)	NA	No response	NA	NA	Acton Prolongatum (1/2); Any (1/2)
Regimen used for ACTH(81)							
Gradually Escalating	24	7	NA	—	NA	NA	—
Maximal dose at initiation	39	5		2			2
Either	2	—		—			—
Initial maximal dose used (48)	75 IU (18/39); 60 IU (10/39); ≤40 IU (9/39); 3 IU/kg and 75 IU/m^2^ (1/39 each)	60 IU (3/5), 75 IU (2/5)	NA	75‐80 IU (2/2)	NA	NA	≤40 IU (2/2)
Frequency of ACTH administration (80)	Once daily (56/64); twice a day (5/64), alternate day (3/64)	Once daily (10/12), twice a day (1/12), alternate day (1/12)	NA	Alternate day (2/2)	NA	NA	Once daily (2/2)
ACTH used in combination with other antiepileptic drugs (81)	55/65	10/12	NA	1/2	NA	NA	2/2
Maximum dose of oral steroids you use (in mg/kg/d) (90)	3‐4 (30/63), 2 (18/63); 5‐8 (13/63); 4‐6 (1/63); 40 mg/d (1/63)	2 (9/12), 3‐4 (1/12); 5‐8 (2/12)	60 mg/d (5/8); 3‐4 (1/8); 5‐8 (1/8); 40‐60 mg/d (1/8)	5‐8 (2/2)	2 (2/2)	60 mg/d (1/1)	2 (2/2)
Duration of treatment for hormonal therapy (91)	4‐12 w (45/64); < 4 w (16/64); >12 w (3/64)	4‐12 w (10/12); < 4 w (2/12)	4‐12 w (5/8); <4 w (3/8	4‐12 w (2/2)	4‐12 w (2/2)	4‐12 w (1/1)	4‐12 w (2/2)
Frequency of blood pressure monitoring advised while on ACTH/oral steroids (91)	Daily (23/65), Weekly (20/65), Alternate day (17/65); Others (5/65)	Weekly (7/11), daily and alternate day (2/11 each)	Daily (4/8), Weekly (3/8), Alternate day (1/8)	Daily or weekly (1/2 each)	Daily or weekly (1/2 each)	Weekly (1/1)	Weekly (2/2)
Frequency of blood/ urine sugar monitoring while on ACTH/ oral steroids (95)	Weekly (34/65), alternate day (12/65), daily (7/65), no monitoring (6/65), others (6/65)	Weekly (7/12), daily (2/12), no monitoring (2/12), alternate day (1/12)	Weekly (3/8), at initiation and end of therapy (3/8), once at 2 w or no monitoring(1/8 each)	Daily or weekly (1/2 each)	Weekly or no monitoring (1/2 each)	Weekly (1/1)	Weekly (2/2)
Prophylactic therapy used after attaining remission with hormonal therapy (92)	33/65	10/12	0/8	0/2	0/2	0/1	1/2
Early relapse (within 1 year) treated with repeat hormonal therapy (92)	48/65	8/12	8/8	2/2	1/2	1/1	2/2

Abbreviations: ACTH, adrenocorticotrophic hormone; d, day; IU, International unit; NA, not applicable; w, weeks.

The maximum dose of oral prednisolone ranged from 2 mg/kg/d to 8 mg/kg/d (Table 2). Most responders from India used 3‐4 mg/kg/d followed by 2 mg/kg/d. Most responders from Pakistan, Bhutan, and Bangladesh used 2 mg/kg/d while those from Sri Lanka, Myanmar, and Nepal used a higher dose of 5‐8 mg/kg/d or 40‐60 mg/d. The duration for which hormonal therapy was administered ranged from 4 to 12 weeks (67/91; 73.6%). Diverse regimes for blood pressure monitoring ranging from daily to weekly were followed throughout India while in Pakistan, most advised weekly monitoring. Weekly monitoring for blood sugar was advised by most responders. Around 48% responders use prophylactic antiepileptic drug after attaining remission with hormonal therapy (all from India, Pakistan, and Bangladesh). Most responders (70/92; 76.1%) treated early relapse (within one year) with repeat ACTH/ oral steroid therapy.

Vigabatrin was used by most responders in all the surveyed nations except Myanmar and Nepal. Rate of escalation of vigabatrin ranged from 25 mg/kg/3 days to 25 mg/kg/wk (Table 3). The maximum duration for which vigabatrin was used ≤9 months (76/99, 76.8%). However, around 25% responders (23/99) used vigabatrin for >9 months duration. Around one‐third of responders do not monitor for ophthalmological problems such as visual field defect while on vigabatrin therapy while the rest routinely advised fundus examination, electroretinogram (ERG), or visually evoked potentials (VEP) for children on vigabatrin therapy.

**TABLE 3:** Management practices pertaining to use of vigabatrin and pyridoxine in West syndrome in the surveyed countries

Question (Responses received)	India	Pakistan	Myanmar	Sri Lanka	Bhutan	Nepal	Bangladesh
Vigabatrin used in practice (123)	70	17	0	5	5	0	2
Rate of escalation for vigabatrin (97)	≥25 mg/kg every 3 d (36/68); every 4‐7 d (23/68); every 2 w (6/68); No hike (3/68)	25 mg/kg/4‐7 d (9/17); ≥25 mg/kg every 3 d (5/17), every 2 w (2/17), No hike (1/17)	NA	25 mg/kg/w (2/5); ≥25 mg/kg /3 d (3/5)	25 mg/kg/3 d (2/5); per w (2/5); per 2 w (1/5)	NA	25 mg/kg/3 d (2/2)
Maximum duration of Vigabatrin therapy (99)	<6 m (30/70), 6‐9 m (25/70), >9 m (15/70)	<6 m (5/17), 6‐9 m (8/17), >9 m (4/17)	NA	<6 m (1/5), 6‐9 m (3/5), >9 m (1/5)	6‐9 m (3/5), >9 m (2/5)	NA	6 m (1/2),> 9 m (1/2)
Monitoring for visual field defect while on vigabatrin therapy (94)	None (23/67), Fundus (14/67), ERG (11/67), VEP (12/67), VF testing (2/67); OCT (2/67); Ophthalmological review (3/67)	Fundus (8/17), None (5/17), VEP (2/17), Ophthalmological review (2/17)	NA	None (5/5)	ERG (2/3), VEP (1/3)	NA	Fundus (1/2), vision assessment (1/2)
Pyridoxine trials used in (119)	n = 74	n = 22	n = 8	n = 6	n = 4	n = 3	n = 2
In all patients	14	15	—	1	—	—	—
In cryptogenic WS before hormonal therapy	30	1	1	1	2	1	—
In cryptogenic WS after hormonal therapy	27	4	6	4	2	1	1
With hormonal therapy	3	2	0	—	—	1	—
Not used/rare	0	2	1	—	—	—	1

Abbreviations: d, day; ERG, electroretinogram; m, months; NA, not applicable; OCT, optical coherence tomography; VEP, visual evoked potential; VF, visual field; w, weeks; WS, West syndrome.

Most responders considered a cessation of spasms for either four weeks (48.6%) or two weeks (41.5%) as a complete response. Most responders advised EEG for evaluating the resolution of hypsarhythmia (104/123; 84.6%). Most responders would proceed with a second‐line drug in cases with failure of clinical response in 14 days (73/121; 60.3%). Around 50% of responders (62/123) had a standard operating protocol for the management of WS at their center. More than 95% of responders felt a need for more awareness regarding WS (Table 1).

### 3.2 Results of systematic review and meta‐analysis

Of 1012 studies screened, 18 studies fulfilled the eligibility criteria and were included for the meta‐analysis (Figure S1).^7^, ^15^, ^16^, ^17^, ^18^, ^19^, ^20^, ^21^, ^22^, ^23^, ^24^, ^25^, ^26^, ^27^, ^28^, ^29^, ^30^, ^31^ These studies were from three countries (India, Pakistan, and Sri Lanka), and there was no published study fulfilling eligibility criteria from Bangladesh, Bhutan, Nepal, and Myanmar. There was substantial heterogeneity among the studies for most variables—gender, LTTT, and structural etiology. However, the heterogeneity was low for the interventional studies testing the effectiveness of hormonal therapy (ACTH or hormonal therapy).

**FIGURE 2:**
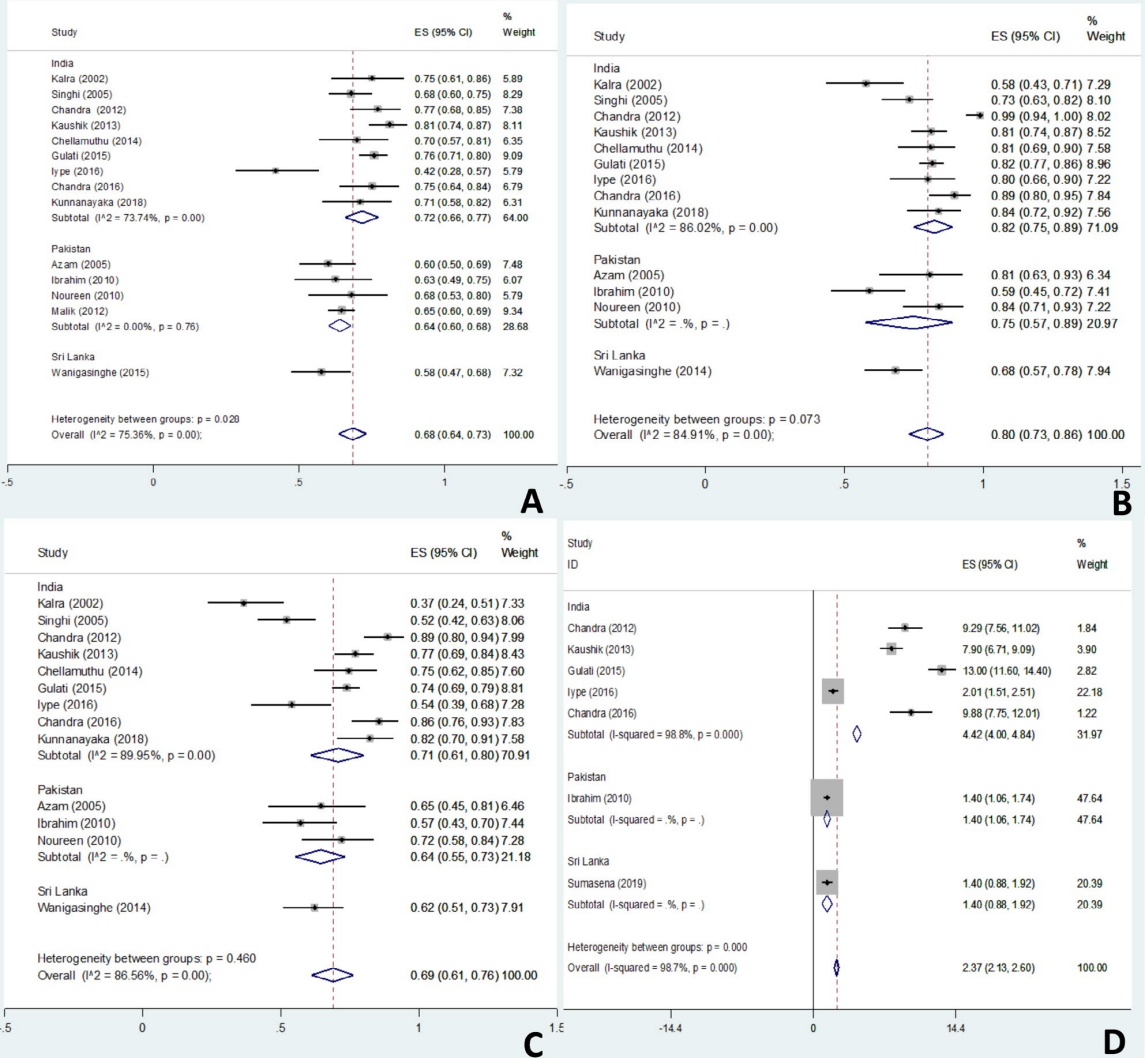
Forest plots for pooled estimates for gender, etiology, and diagnostic or treatment lag in children with West syndrome. Forest plots showing the South Asian and country‐wise pooled estimates for the proportion of male gender (A), structural etiology (including intrauterine infections, malformations, and neurocutaneous syndromes) (B), acquired structural insult (including intrauterine infections; excluding malformations) (C), and lead time to diagnosis or treatment (in months; D) in children with West syndrome

The pooled estimates for proportion of male gender, structural etiology, and acquired structural insult revealed preponderance of these variables [male gender: 68% (confidence interval [CI] 64%‐73%), structural etiology: 80% (CI 73%‐86%), acquired structural insult: 69% (CI: 61%‐76%)] in the included studies (Figure 2A,B,C). For male gender, the pooled estimates varied among different countries from 58% in Sri Lanka to 72% in India.^15^, ^16^, ^17^, ^18^, ^19^, ^20^, ^21^, ^22^, ^23^, ^29^ The pooled estimate for LTTT was 2.4 months (CI: 2.1‐2.6 months) while for India, it was nearly two times [4.4 months (CI: 4‐4.8 months)] (Figure 2D).^17^, ^18^, ^20^, ^21^, ^22^

**FIGURE 3:**
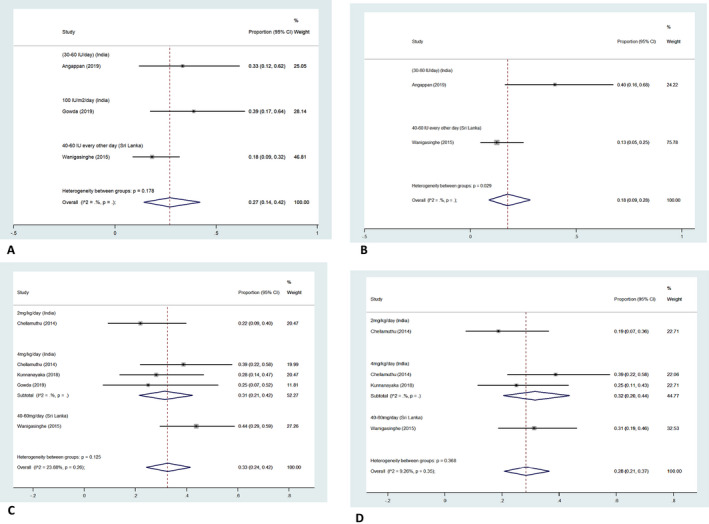
Forest plot showing the pooled estimates for response to hormonal therapy [adrenocorticotropic hormone (ACTH) and oral steroids] in children with West syndrome in South Asia. Forest plot showing the pooled estimates for electroclinical response at day 14 and persistent cessation of spasms at day 42 (among patients with freedom of spasms/ electroclinical response at day 14) of ACTH (A and B) and oral steroids (C and D) in South Asian children with West syndrome

For the effectiveness of hormonal therapy, limited number of interventional trials was available.^7^, ^19^, ^23^, ^29^, ^31^ These studies were from India and Sri Lanka. These studies used different dose regimes for ACTH and oral steroids, although the preparations were same, that is, Acton Prolongatum and prednisolone. The pooled estimates for electroclinical response at day 14 and persistent cessation of spasms at day 42 of ACTH therapy were 27% (CI: 14%‐42%) and 18% (CI: 9%‐28%) while those for oral steroid were 33% (CI: 24%‐42%) and 28% (CI: 21%‐37%), respectively (Figure 3A‐D). For ACTH, the diamond was significantly skewed toward the Sri Lankan study (with a lower response rate, larger sample size, and hence weight).^29^ However, ACTH was used every other day in this study, and the dose was nearly half of that used in Indian studies.^7^, ^31^

## 4 DISCUSSION

The current study brings forth management practices for WS in seven South Asian countries. A response rate of 56% was observed for the survey. This was much higher as compared with a survey of Child Neurology Society members (USA; 18%) while this was low as compared with a survey in Japan (75%), probably due to wider regions covered.^12^, ^13^ It was observed that there is a wide diversity in the practices, and each country has its own challenges.

Most responders observed a male preponderance for WS. This concurs with the results of meta‐analysis with a pooled male to female ratio of 2.1:1. However, the predilection for male gender varied among the countries in the meta‐analysis with a pooled male to female ratio of 2.6:1 for India, 1.8:1 for Pakistan, and 1.4:1 for Sri Lanka (Figure 2A).^15^, ^16^, ^17^, ^18^, ^19^, ^20^, ^21^, ^22^, ^23^, ^24^, ^25^, ^26^, ^27^, ^29^ Although slight male preponderance has also been reported globally (male: female‐ 1.5:1), the figures for South Asia (especially India) are definitely higher.^2^, ^11^ This disparity may be explicated by the higher risk of perinatal complications such as intrauterine growth restriction, hypoxic‐ischemic encephalopathy (HIE), and hypoglycemia in male infants.^11^, ^32^ Another reason may be a higher proportion of males in the referred population.^2^

Most responders observed a longer LTTT (4‐12 weeks) and the predominance of structural etiology. Interestingly, some responders from Pakistan observe genetic causes more commonly, probably due to higher consanguinity. These responses are in accord with the results of meta‐analysis with a pooled mean LTTT of 2.4 months and a static structural insult to the tune of 80%.^15^, ^16^, ^17^, ^18^, ^19^, ^20^, ^21^, ^22^, ^23^, ^24^, ^25^, ^26^, ^29^, ^30^ The longer LTTT estimate (4.4 months) and a higher preponderance of static structural insult (82%) and acquired structural insult (71%) in India are bothersome. The LTTT is relatively short in Western countries (<4 weeks) and is known to be the most important prognostic factor in WS.^5^ Both these factors—a longer LTTT and a high proportion of static structural insult—may adversely affect the developmental outcomes for WS in South Asia. Acquired brain insult secondary to HIE, hypoglycemic brain injury, etc is fairly common in developing countries and is a common cause of WS.^15^, ^16^, ^17^, ^18^, ^19^, ^20^, ^21^, ^22^, ^23^, ^24^, ^25^, ^26^, ^29^, ^30^ Despite the growing focus on perinatal and neonatal health, substantial mortality and morbidity persists and a significant proportion of this is caused by preventable causes such as birth asphyxia and neonatal hypoglycemia. Thus, there is a growing need to prioritize the resources and research strategies at avoiding the preventable causes and reducing the diagnostic lag and thereby LTTT.

Lack of event recognition as seizure by parents was considered as the most common cause of treatment lag followed by misdiagnosis by the initial attending physician. This highlights the poor awareness not only among the parents but also among the practitioners.^14^, ^18^, ^20^ Hence, there is an unmet need for creating awareness regarding WS. Home video recordings or video recordings by pediatricians may be helpful in early diagnosis and reducing LTTT. Also, the scarcity of pediatric EEG facilities in Bhutan and Nepal is worrisome. Although there is provision for free diagnosis and management in Bhutan, this cannot recompense the lack of infrastructure for diagnosis.

Akin to the rest of the world, hormonal therapy remains the mainstay of treatment. However, there is an inclination toward oral steroids in comparison with ACTH. This might be attributed to several reasons. These include nonavailability of ACTH in Myanmar, Nepal, and Bhutan, cost of ACTH therapy, expenses being borne by patient families in most South Asian countries (instead of state as in the western countries), painful and cumbersome frequent injections of ACTH, mounting evidence for efficacy of oral steroids from South Asia, etc.^24^, ^29^, ^30^ This is also apparent in the meta‐analysis where the electroclinical response rates on day 14 and persistent cessation of spasms on day 42 of ACTH and prednisolone therapy favor oral prednisolone. These response rates are much lower than those seen in Western countries. The possible reasons for this discrepancy include the preponderance of structural etiology and a longer LTTT. Also, the results of meta‐analysis are predominantly based on the largest trial from South Asia by Wanigasinghe et al which used an alternate‐day regime for ACTH as compared with other Indian studies which reported response rates nearly two times of those reported in the Sri Lankan study.^29^

Different forms of ACTH are used in WS. These include natural ACTH (short‐acting form derived from bovine or porcine source; used in the USA) and synthetic ACTH (such as tetracosactide used in Europe and Asia; Acton prolongatum in Asia, etc).^33^ Natural ACTH (derived from animal sources) consists of 39 amino acids with corticotrophic activity in initial 20 amino acids at N‐terminal. The antigenic activity lies in the C‐terminal portion (22‐39 amino acids) of the molecule. Synthetic ACTH may contain all 39 amino acid residues (Acton Prolongatum‐porcine sequence) or lesser amino acids with biological activity (tetracosactide, cosyntropin, synacthen, etc containing 24 amino acids). Also, these are usually constituted in depot form (Synacthen) or in combination with carboxymethyl cellulose (Acton Prolongatum) to allow for a longer duration of action.

The most commonly used preparation for ACTH in the seven surveyed countries was Acton prolongatum (a synthetic ACTH with porcine sequence). Natural ACTH is not available. Although there are no head‐to‐head clinical trials comparing natural and synthetic ACTH, Acton Prolongatum is a cheaper (as compared with natural ACTH or Synacthen). The most commonly followed regime was maximal‐dose‐at‐initiation (60‐75 IU or 150 IU/m^2^). The rate of administration ranged from once daily in most countries to alternate day in Sri Lanka. Alternate‐day tetracosactide administration has also been in practice in the UK, considering higher side effects with daily administration.^34^ However, there are no head‐to‐head trials comparing daily with alternate‐day administration and gradually escalating regime with maximal‐dose‐at‐initiation regime. For oral steroids, United Kingdom Infantile Spasms Study (UKISS) regime is followed in Sri Lanka, Myanmar, and Nepal while a dose of 2‐4 mg/kg/d is used in India, Pakistan, Bangladesh, and Bhutan.

Vigabatrin was used by most responders in all the surveyed nations except Myanmar and Nepal. However, there are issues with licensing and availability of the drug in most of the surveyed countries. The drug available is usually imported, thereby adding to the cost of therapy. It is vexing that neither ACTH nor vigabatrin are licensed and available in Nepal and Myanmar while ACTH is not licensed and available in Bhutan. Vigabatrin is neither licensed and nor readily available in India. Around 20% of responders used vigabatrin for >9 months, which is known to predispose to visual field defects.

Standardized management protocols are known to be associated with better outcomes in WS.^35^ Hence, there is a need for the development of a standardized protocol for the management of WS at each center. Besides this, there is a need for consensus guidelines for South Asian subcontinent, considering the unique problems faced by these countries. Many of the problems which are common to these countries such as longer LTTT and structural etiology may be targeted as a group. However, despite several commonalities, establishing a unified protocol for management in these countries may be difficult considering the lack of proper facilities for investigations like EEG in some countries, availability issues with ACTH and vigabatrin in most of these countries, and probably different response rate of different populations to oral steroids and ACTH.

The current study is a singular study from South Asia on the management practices for WS, highlighting various challenges and providing a meta‐analysis of available literature. Also, this study underscores the remediable problems such as difficult access to pediatric EEG, availability and licensing issues for drugs used in WS in many South Asian countries. Solutions to these may improve the outcomes of children with WS in South Asia.

This study was limited to a survey of the professionals interested in the field. Besides, only two pediatric neurologists could be contacted in Bangladesh, and the response rate to the survey was around 50% in India (despite a high number) and Bhutan. Hence, this study may not be the most accurate depiction of the practices in these countries. But the concurrence between the results of survey and the meta‐analysis suggests exactitude of the survey results.

## 5 CONCLUSION

This survey brings to light the practices and challenges in the management of WS in South Asia. The important practices comprise a preference of longer acting synthetic ACTH in India and Bangladesh, a preference for oral steroids/vigabatrin over ACTH in Sri Lanka, Nepal, and Bhutan, and wide variations in treatment regimens for ACTH, oral steroids, and vigabatrin across the region. The challenges include a longer LTTT, a preponderance of structural etiology, difficult access to pediatric EEG, and nonavailability of ACTH and vigabatrin in some countries. With teeming numbers of children with WS in these countries, pediatric neurologists should take these challenges as an opportunity for liaison for advocacy, develop a standardized management protocol, and conduct research to develop affordable and sustainable healthcare solutions to improve the long‐term outcome in WS.

## CONFLICT OF INTEREST

Dr Jitendra Kumar Sahu serves as a section editor for Indian Journal of Pediatrics and received project grant from Indian Council of Medical Research for “West syndrome‐EAST" trial, however, no disclosure pertaining to the study. Other authors report no disclosures. We confirm that we have read the Journal's position on issues involved in ethical publication and affirm that this report is consistent with those guidelines.

## AUTHORS' CONTRIBUTION

Priyanka Madaan contributed by planning the study, literature search, data collection, data analysis, and writing the manuscript. Prem Chand contributed by data collection in Pakistan, data analysis, and critical review of the manuscript for intellectual content. Kyaw Linn contributed by data collection in Myanmar, data analysis, and critical review of the manuscript for intellectual content. Jithangi Wanigasinghe contributed by data collection in Sri Lanka, data analysis, and critical review of the manuscript for intellectual content. Mimi Lhamu Mynak contributed by data collection in Bhutan, data analysis, and critical review of the manuscript for intellectual content. Prakash Poudel contributed by data collection in Nepal, data analysis, and critical review of the manuscript for intellectual content. Raili Riikonen contributed by inputs in designing the study, data analysis, and critical review of the manuscript for intellectual content. Amit Kumar contributed by study search for meta‐analysis, data analysis, and critical review the manuscript for intellectual content. Pooja Dhir contributed by data analysis and critical review of the manuscript for intellectual content. Sandeep Negi contributed by planning the study, data collection, data analysis, and critical review the manuscript for intellectual content. Jitendra Kumar Sahu contributed by conception and planning the study, data analysis, data interpretation, and writing of the manuscript. All authors approved the final version of manuscript to be published and agreed to be accountable for all aspects of the work in ensuring that questions related to the accuracy or integrity of any part of the work are appropriately investigated and resolved. Statistical analysis: It was completed by SN, PM, and AK.

## Supporting information

Supplementary Material
