# Supplementary material for: Management practices for West syndrome in South Asia: A survey study and meta‐analysis
Source: Epilepsia Open. 2020 Aug 11;5(3):461–74. doi: 10.1002/epi4.12419 (PMC7469760; doi:10.1002/epi4.12419)
Supplement: Supplementary file 1 — Supplementary Material [file EPI4-5-461-s001.docx]

**Supplementary table 1 Detailed methodology of systematic review and meta-analysis**

|  | **Detailed methodology** |
| --- | --- |
| 1. Search Strategy | - *Search terms*: “West syndrome” OR “Infantile spasms” AND “South Asia” OR “India” OR “Pakistan” OR “Myanmar” OR “Nepal” OR “Bangladesh” OR “Afghanistan” OR “Maldives” OR “Sri Lanka” OR “Bhutan” - *Databases*: PubMed, Google Scholar - *Date*: November 2019 |
| 1. Selection of studies | ***For epidemiological variables*** (gender preponderance, etiology, lead time to diagnosis/ treatment)  *Inclusion criteria*: Observational and interventional studies with >50 patients with WS from South Asia (India, Pakistan, Sri Lanka, Bangladesh, Nepal, Bhutan, and Myanmar)  *Exclusion criteria*:   - Observational studies with less than 50 patients - Data on same study population   ***For response rate to hormonal therapy***  *Inclusion criteria*: Randomized controlled trials with oral steroids or ACTH as intervention  *Exclusion criteria*: Relevant outcome not reported  Done by 2 authors: PM and JSK |
| 1. Data analysis | The data analysis was performed using the STATA version 13  software. The random effect model with Freeman-Tukey double arcsine transformation and exact confidence intervals for the individual studies were used to analyze the data. |
| 1. Variables and number of studies for each variable | - Gender (preponderance of male gender) - Etiology - Structural etiology (including intrauterine infections, malformations, and neurocutaneous syndromes) - Acquired structural insult (including intrauterine infections; excluding malformations) - Lead time to diagnosis or treatment: Includes time from onset of the spasms to diagnosis (lead time to diagnosis; LTTD) or treatment (lead time to treatment; LTTT). LTTT was used for the purpose of analysis for studies where both were available. - Response rate to ACTH and oral steroids - Electroclinical response at day 14 and - Persistent cessation of spasms at day 42 of therapy among patients with spasm freedom/ electroclinical response at day 14 |

**Supplementary figure 1. PRISMA diagram**

12 of full-text articles excluded

- Observational studies with less than 50 patients: 10
- Data on same study population: 2

946 of records screened for titles and abstracts

30 of full-text articles assessed for eligibility

18 of studies included in quantitative synthesis (meta-analysis)

## Identification

## Included

## Eligibility

1009 of records identified through database searching (PubMed, Google Scholar)

916 of records excluded

66 of duplicates removed

3 of additional records identified through other sources

## Screening
